# Fucoidans of Brown Algae: Comparison of Sulfated Polysaccharides from *Fucus vesiculosus* and *Ascophyllum nodosum*

**DOI:** 10.3390/md20100638

**Published:** 2022-10-13

**Authors:** Anatolii I. Usov, Maria I. Bilan, Nadezhda E. Ustyuzhanina, Nikolay E. Nifantiev

**Affiliations:** The Laboratory of Glycoconjugate Chemistry, N.D. Zelinsky Institute of Organic Chemistry, Russian Academy of Sciences, Leninsky Prospect 47, 119991 Moscow, Russia

**Keywords:** brown algae, *Fucus vesiculosus*, *Ascophyllum nodosum*, fucoidans

## Abstract

Preparations of sulfated polysaccharides obtained from brown algae are known as fucoidans. These biopolymers have attracted considerable attention due to many biological activities which may find practical applications. Two Atlantic representatives of Phaeophyceae, namely, *Fucus vesiculosus* and *Ascophyllum nodosum*, belonging to the same order Fucales, are popular sources of commercial fucoidans, which often regarded as very similar in chemical composition and biological actions. Nevertheless, these two fucoidan preparations are polysaccharide mixtures which differ considerably in amount and chemical nature of components, and hence, this circumstance should be taken into account in the investigation of their biological properties and structure–activity relationships. In spite of these differences, fractions with carefully characterized structures prepared from both fucoidans may have valuable applications in drug development.

## 1. Introduction

Sulfated polysaccharides containing L-fucose as the main monosaccharide component were discovered in several brown algae, including *Fucus vesiculosus* and *Ascophyllum nodosum*, more than a century ago [1]. Now, it is well known that preparations obtained by extraction of algae and designated by trivial name “fucoidans” usually represent complex mixtures of several chemically different polysaccharides [2,3,4], where fucan sulfate (FS, a polysaccharide built up of fucose and sulfate only) may (but not necessarily) be one of the main components. The procedure suitable for commercial production of fucoidans from brown algal biomass was suggested in 1952 [5], but a highly purified sample of more or less individual FS was obtained much later by numerous manipulations with crude fucoidan from *F. vesiculosus*, probably accompanied by considerable loss of the starting material [6]. The first data on the chemical structure of SF published in 1950 [7,8] were then revised several times. In this period, evidence appeared on the prospective biological activity of fucoidans, especially as anticoagulants [9,10].

The availability of commercial fucoidans resulted in the rapid appearance of a large amount of publications devoted mainly to the investigation of their biological properties [11]. At present, there are hundreds of such papers; for example, more than 400 references dedicated mainly to therapeutic applications of fucoidans were cited in three reviews by Fitton et al. [12,13,14]. Multiple biological actions of fucoidans depend primarily on their interaction with different proteins due to the presence of sulfate groups [15,16,17,18,19], but branching of molecules [20] and molecular weights [21,22] may also be very important factors. Fucoidans are traditionally regarded as promising anticoagulant [23,24,25,26], antitumor [27,28] and anti-inflammatory agents [29,30,31,32,33], but recently they acquired special importance as potential components of antiviral drugs [34,35,36], activators of hematopoiesis [37,38], and reagents for use in nanomedicine [39].

The elucidation of correlations between the biological properties of concrete samples of fucoidans and their chemical structures remains a very important task [23,40], but there are substantial difficulties connected with the conception of “fucoidan”, which is not the term of strict carbohydrate nomenclature. It designates a preparation of water-soluble sulfated polysaccharides, obtained by extraction of brown algal biomass and separated (partially or completely) from other polysaccharide components of this biomass devoid of sulfate groups—alginates and laminarans. The class Phaeophyceae (brown algae) numbers more than 1000 species, which may differ considerably in their polysaccharide composition [41,42]. Thus, fucoidans isolated from different species may contain not only fucopyranose, but also fucofuranose units [43] together with other monosaccharides, such as galactose, xylose, mannose, and glucuronic acid, etc. [2,3,4,41].

Therefore, the algal species is the first factor determining the composition of extracted sulfated polysaccharides. Then it is necessary to bear in mind that representatives of the same species growing in different conditions should inevitably differ in chemical composition. Ecological factors influencing the chemical composition of biomass include age and physiological status of the alga [44], climate and season [45], water temperature and salinity, solar radiation, and the accessibility of biogenic elements. Additional influences on the quality of polysaccharide preparations should have the procedures of harvesting and storage of raw material, as well as conditions of biomass treatment, which should secure the completeness of polysaccharide extraction without their degradation and minimal dissolution of extraneous non-carbohydrate materials, such as proteins and polyphenols. Since crude preparations of sulfated polysaccharides, obtained by water extraction, are usually mixtures of biopolymers of different structures [46], the nature of products destined for structural analysis or the investigation of biological properties will be determined by the used methods of fractionation [47]. Taking into account all the factors listed above, it is not surprising that different groups of researchers, dealing with samples being commonly named by the same term “fucoidan”, in fact are often working with very different polysaccharides [48].

Below we overview the published results of structural studies of polysaccharides obtained from two widely distributed brown algal species, namely, *F. vesiculosus* and *A. nodosum*. Both these species have been investigated for a long time and may be regarded as prospective sources for the large-scale preparation of sulfated polysaccharides suitable for diverse medical applications [49]. For example, based on such polysaccharides, biological vectors are being developed, which may be used for delivering drugs or diagnostic contrasting agents to tissues with increased P-selectin expression [50,51,52,53,54]. It should be emphasized that GMP-graded production of low-molecular-weight fucoidan from *A. nodosum* was recently reported [54], and it was suggested to apply this product as the biovector or contraster for the detection of P-selectin expression during cardiovascular diseases.

## 2. Sulfated Polysaccharides of *Fucus vesiculosus*

Representatives of the genus *Fucus* are widely distributed in the North Atlantic, as well in the Barents and the Whyte Seas. Being typical littoral species, they occupy spacious coastal plots uncovering at the low tide. Harvesting of this natural raw material is not a very difficult task.

*F. vesiculosus* was one of those several brown algal species wherein the presence of fucoidans was discovered [1]. Polysaccharide preparation from this species was used in the first attempt to elucidate its chemical structure [7,8]. The alga was heated with water in the boiling water bath, the extract obtained was treated with lead acetate to remove alginic acid and proteins, and the remaining soluble polysaccharides were reprecipitated several times and yielded a preparation, which was regarded as a fucan sulfate. According to the analytical data, an essentially linear structure was suggested for the backbone of this polysaccharide, built up of 1→2-linked α-L-fucopyranose residues, together with some unsubstituted or monosulfated fucose residues attached to position 3 of the backbone as single branches.

**Figure 1:**
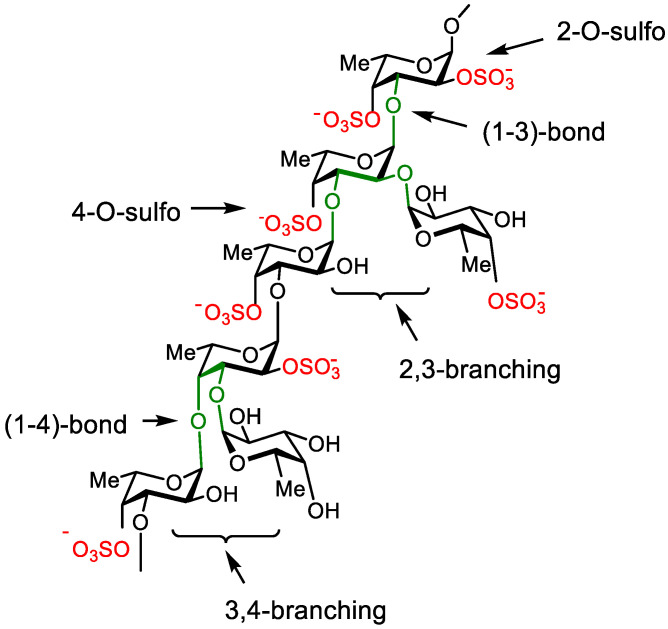
The current idealized formula of brown algal fucan sulfate (structural elements, which are possibly important for biological activity, are indicated).

About 40 years later, a commercial fucoidan from *F. vesiculosus* was reinvestigated and the structure of hypothesized fucan sulfate was corrected [55]. It was shown that 1→3-linkage is the main type of linkage between the backbone residues. Possible positions of sulfate and branches are depicted in Figure 1. Similar figures are often used to illustrate structures of FS isolated from other brown algal species [56]. It should be noted that this formula does not belong to any concrete sample of FS, but shows only the set of different units, which may be found in different proportions in different samples of polysaccharides.

The structure of the backbone was revised once more after the introduction of NMR spectroscopy in the practice of structural analysis of FS. Spectral data gave reliable confirmation on the presence of 1→3-linked backbone in FS isolated from *Chorda filum* [57] and several other algae belonging to the order Laminariales, but showed that fucoidans from *F. vesiculosus* and *A. nodosum* contain fractions with backbones built up of alternating (1→3)- and (1→4)-linked fucose residues [58,59]. Polysaccharides containing two alternating linkages in the backbones were also found in several other representatives of the order Fucales [60,61,62]. A hypothesis once appeared that (1→3)-linked FS backbones are characteristic for algae from Laminariales only, whereas backbones with alternating (1→3)- and (1→4)-linkages are typical for algae from Fucales, but the existence of such a firm correlation between the taxonomic position of the algae and the structure of their FS was not confirmed in recent investigations [48].

The formula of FS depicted in Figure 1 does not take into account the presence of several other monosaccharides, primarily galactose, xylose, mannose, and glucuronic acid, which usually may be found in fucoidan preparations. These monosaccharides may be components of other types of polysaccharides. According to contemporary evidence, several polysaccharides forming brown algal cell walls are linked with proteins and polyphenols in a complex [4], where the nature of linkages between components remains mostly unknown. To isolate polysaccharides, this complex should be destroyed by, for example, the action of dilute acids [5], although FS itself may be partially degraded under acid conditions. Crude fucoidans usually contain a wide set of molecules differing in composition and molecular weights and evidently need additional purification. Anion-exchange chromatography demonstrates the presence of continuous spectrum of molecules differing in charge and monosaccharide composition, from fractions with low sulfate and low fucose, containing other neutral monosaccharides and glucuronic acid, to highly sulfated fucans [63]. Simultaneous presence of several different sulfated polysaccharides was demonstrated for many brown algae [46,64]. The isolation of the components of these mixtures depends on their relative content and specific extraction and fractionation procedures. More detailed discussion on the problem is given below in the description of polysaccharides from *A. nodosum*.

One of the laboratory procedures of fucoidan isolation [60] recommends extraction of algal biomass with 2% aqueous calcium chloride at 85 °C. It results in dissolution of neutral laminaran and sulfated polysaccharides, whereas insoluble Ca-salts of alginic acids remain in the precipitate. Acid polysaccharides may be precipitated from extract by the addition of a cationic detergent, such as cetyltrimethylammonium bromide, transformed into the soluble sodium salts and then chromatographed on an anion-exchanger, such as DEAE-Sephacel, using stepwise elution with NaCl solutions of increasing concentrations. Neutral components are not absorbed on the column, while alginic acids are eluted with 0.5 M NaCl, and sulfated fractions are then eluted according to the increase of sulfate content, the most sulfated fractions appearing in the region of 2.0 M NaCl.

There are numerous modifications of isolation procedure aimed at acceleration of the process or increase of the yield of target polysaccharides. For these purposes it was suggested to isolate cell walls [64,65], to carry out extraction with solutions of acids, alkali, or detergents [64,66,67], to use autohydrolysis [68], microwave radiation [69], ultrasound [70], or treatment with enzymes capable of destroying the accompanying polysaccharides [71,72]. Fucoidans prepared by different procedures may differ considerably in composition and properties. Thus, seventeen fucoidans isolated by different authors from *F. vesiculosus* contain from 4% to 39% of sulfate and from 50% to 94% of fucose in carbohydrate moiety. These analytical characteristics were given in a recent review [48].

## 3. Sulfated Polysaccharides of *Ascophyllum nodosum*

This brown algal species belonging to the same family Fucaceae, as described above *F. vesiculosus*, practically coincides with it in geographical distribution, but is growing in sublittoral conditions. *A. nodosum* is used for industrial production of alginates and is available as feedstock in large amounts.

A peculiar sulfated heteropolysaccharide named “ascophyllan” was isolated from the mixture of water-soluble polysaccharides obtained by extraction of *A. nodosum* [73]. It contained approximately equimolar amounts of fucose, xylose, glucuronic acid, and sulfate, as well as a tightly bound polypeptide fragment. Partial acid hydrolysis of ascophyllan gave rise to 3-O-β-D-xylopyranosyl-L-fucose [74] and a non-dialysable polyuronide, indicating the presence of a backbone built up of glucuronic acid residues and side chains containing fucose and xylose. Using extractions under very mild conditions, it was possible to isolate a high-molecular complex, which was split under subsequent acid treatment into ascophyllan, alginate, and a fraction close to fucan sulfate in composition. Based on these data, it was supposed that a similar situation should also be found for polysaccharides of *F. vesiculosus*, but in this case the content of fucan sulfate predominates considerably over the content of hypothetical ascophyllan analogue [75]. Structural analysis of ascophyllan was the subject of a series of more recent publications [76,77,78,79,80,81]. Based on these data, a procedure was developed for isolation of a fraction of fucan sulfate from *A. nodosum*, having backbones of 1,3-linked fucose residues [82]. At the same time another fraction, containing backbones of alternating 1,3-1,4-linked fucose residues, was obtained from the same alga by another group of authors. The structure of the latter polysaccharide was carefully investigated using chemical methods together with NMR spectroscopy [58,59] and mass spectrometry [83]. Hence, fucan sulfate itself is heterogeneous and contains fractions having fundamental structural differences in their carbohydrate moieties.

Later on, a simplified procedure of biomass treatment was published, which gives the possibility to prepare ascophyllan and fucan sulfate separately [84] (Table 1). Polysaccharides isolated by this procedure were studied in biological tests, and it was found for the first time that ascophyllan can stimulate the growth of a culture of mammalian cells (under the same conditions fucan sulfate showed opposite action) [85]. Comparison of polysaccharide preparations, isolated from *A. nodosum* by usual extraction with dilute acid, and by several new procedures using microwave radiation [86], ultrasound, or enzymatic degradation of cell walls was described in a recent paper [87]. As expected, these preparations differ in yields, composition, and molecular weights, but have comparable prebiotic activity by stimulating, in vitro, the growth of lactic acid bacteria. Two polysaccharide samples differing in molecular weights and capable of inhibiting inflammation were isolated by treatment of *A. nodosum* with enzymes followed by anion-exchange chromatography [88]. Both preparations contained not only fucose, but also galactose and hence were fragments of a sulfated galactofucan. They exhibited different anti-inflammatory activities, indicating that molecular weight is an important factor for this type of biological action.

## 4. Conclusions

Both brown algal species described in this review are convenient sources of so-called “fucoidans”, which are crude mixtures of sulfated polysaccharides. Both “fucoidans” contain fucan sulfates as the main components, which are especially interesting as biologically active polysaccharides. Differences between “fucoidans” of these two algal species are connected mainly with the higher content of another main component, termed “ascophyllan”, in *A. nodosum*. In fact, ascophyllan itself is a mixture of several heteropolysaccharides of moderate sulfation degree, containing, in addition to fucose, also xylose, glucuronic acid, and some other monosaccharides [73,77,81]. Ascophyllan has its own practically useful properties and may find application as a preparation with peculiar (distinct from fucan sulfates) biological activities [89]. The paper by W. Jin et al. [90] may be mentioned as a very impressive example of investigation devoted to correlating the biological activity and chemical structures of fucoidan components: the authors carefully analyzed the structural information of about sixty fucoidan samples isolated from different algae, used them in a cell surface tau-binding assay, and found that two different branched sulfated polysaccharide components of fucoidans, namely, a galactofucan and a fucoglucuronomannan, acted as effective inhibitors of tau spreading. Hence, these data may provide a basis for creation of drugs applicable for therapy in the earliest phase of Alzheimer’s disease.

Resolution of “fucoidans” into individual polysaccharide components remains the most difficult problem. Since these polysaccharides are polyanions, anion-exchange chromatography is traditionally used for their separation, giving excellent results in some cases [61]. At the same time this procedure cannot resolve compounds differing in structure, but having similar charge densities. Similar limitations are typical for gel-permeation chromatography, where even structurally different polysaccharides cannot be resolved, if they have close molecular weights. Evidently, the resolution of fucoidans needs additional improvement. Specific enzymatic degradation of unnecessary components of the mixture [91] or affinity chromatography based on the property of sulfated polysaccharides to bind several proteins [92,93] may be mentioned as the possible approaches to new fractionation procedures.

Fucoidan preparations devoted to medical applications should satisfy the very strong demands concerning their reproducible composition and compatibility with manufacturing requirements under GMP-standards. A similar problem has been solved in the preparation of low-molecular-weight heparins [94]. The process of fucoidan isolation should be carefully controlled at several steps. One of the most important parameters is the standard quality of the raw material, which is highly varying and depending on the place and season of harvesting, as well on the procedure of its conservation and storage. Extraction may be carried out with dilute acids under moderate heating at the conditions, which are sufficient for the destruction of polysaccharide complexes of the cell walls without marked degradation of the target fucoidan. Mild oxidants may be used for bleaching, and high molecular mass may be diminished (if necessary) by careful partial depolymerization. The wanted fraction may be prepared using membrane filtration. Finally (this is especially important for algae similar in polysaccharide composition to *A. nodosum*) the most interesting biologically active highly sulfated fraction should be separated from lower-sulfated material (such as so-called ascophyllan) using chromatography on anion-exchange resins. The preparations obtained should be characterized by quantitative determination of monosaccharides (e.g., gas–liquid chromatography, spectrophotometry) sulfate (e.g., turbidimetry) and molecular-mass distribution (e.g., analytical gel-permeation chromatography). Similar standardization procedures are suggested for sulfated glycosaminoglycans, such as, for example, for chondroitin sulfates [95].

Regulatory requirements mentioned above shorten the fields of medical use of fucoidans by the case of superficial applications (ointments, gels, inhalable compositions, etc.), but injectable forms may be based on synthetic oligosaccharides related to fucoidan fragments. It should be emphasized that the preparation of such compounds is well elaborated to date [96,97,98]. In addition, one promising option for fucoidan standardization can be connected with enzymatic treatments. Recent studies discovered a series of fucoidan degrading and modifying enzymes [99,100,101], but up to now only a few agents of this type were obtained, while their applicability for industrial application was not studied yet and needs to be investigated.

## Figures and Tables

**Table 1:** Yields and composition (in %) of several polysaccharide preparations isolated from *A. nodosum* and *F. vesiculosus* *.

Preparation	Yield	Fuc	Xyl	Glc	Man	Gal	UA	SO_3_Na
Ascophyllan [71]	1.9	15.5 (1.00)	13.4 (0.95)	0.3 (0.02)	3.4 (0.2)	0.6 (0.04)	21.4 (1.17)	9.6 (1.06)
Fucan sulfate from *A. nodosum* [71]	1.25	28.4 (1.00)	4.3 (0.16)	2.0 (0.06)	0.8 (0.03)	5.3 (0.17)	5.8 (0.17)	19.4 (1.17)
Fucan sulfate from *A. nodosum* [69]		(1.00)	(0.05)	-	-	-	-	(0.47)
Fucan sulfate from *F. vesiculosus* [5]		(1.00)	tr.	-	-	tr.	-	(0.47)
Commercial fucoidan from *F. vesiculosus* [71]		24.8 (1.00)	1.9 (0.09)	0.8 (0.03)	1.0 (0.04)	3.1 (0.11)	9.6 (0.33)	22.6 (1.56)

* Molar proportions relative to fucose content (Fuc = 1.00) are given in brackets.

## Data Availability

Data are available from the corresponding author.
